# Pharmacological Treatment of Anxiety Disorders: The Role of the HPA Axis

**DOI:** 10.3389/fpsyt.2020.00443

**Published:** 2020-05-15

**Authors:** Gustavo E. Tafet, Charles B. Nemeroff

**Affiliations:** ^1^Department of Psychiatry and Neurosciences, Maimónides University, Buenos Aires, Argentina; ^2^Department of Psychiatry, University of Texas at Austin, Austin, TX, United States

**Keywords:** pharmacology, anxiety, hypothalamic-pituitary-adrenal, corticotropin releasing hormone, stress

## Abstract

Stress in general, and early life stress in particular, has been associated with the development of anxiety and mood disorders. The molecular, biological and psychological links between stress exposure and the pathogenesis of anxiety and mood disorders have been extensively studied, resulting in the search of novel psychopharmacological strategies aimed at targets of the hypothalamic-pituitary-adrenal (HPA) axis. Hyperactivity of the HPA axis has been observed in certain subgroups of patients with anxiety and mood disorders. In addition, the effects of different anti-anxiety agents on various components of the HPA axis has been investigated, including benzodiazepines, tricyclic antidepressants (TCAs), and selective serotonin reuptake inhibitors (SSRIs). For example, benzodiazepines, including clonazepam and alprazolam, have been demonstrated to reduce the activity of corticotrophin releasing factor (CRF) neurons in the hypothalamus. TCAs and SSRIs are also effective anti-anxiety agents and these may act, in part, by modulating the HPA axis. In this regard, the SSRI escitalopram inhibits CRF release in the central nucleus of the amygdala, while increasing glucocorticoid receptor (GRs) density in the hippocampus and hypothalamus. The molecular effects of these anti-anxiety agents in the regulation of the HPA axis, taken together with their clinical efficacy, may provide further understanding about the role of the HPA axis in the pathophysiology of mood and anxiety disorders, paving the way for the development of novel therapeutic strategies.

## Introduction

**Figure 1:**
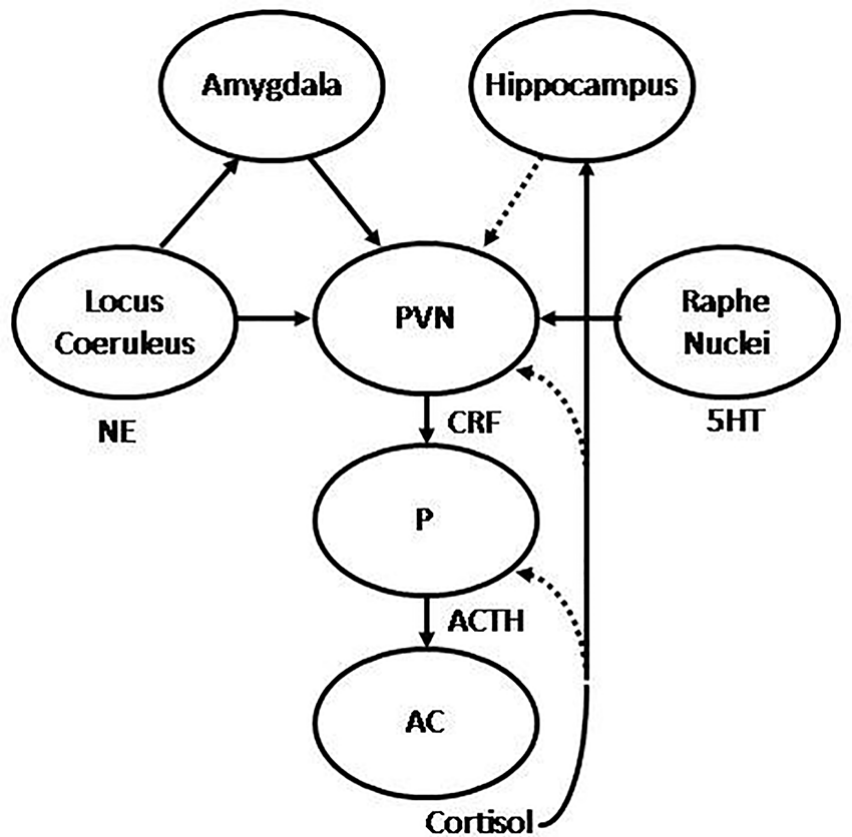
Graphic representation of the hypothalamic-pituitary-adrenal (HPA) axis. The hypothalamic paraventricular nucleus (PVN) releases corticotropin releasing hormone (CRH) to the hypophyseal portal blood, to reach the pituitary (P), where adrenocorticotropic hormone (ACTH) is synthesized and released to systemic blood to reach the adrenal cortex (AC), where in turn cortisol is synthesized and released to the main bloodstream. The HPA is regulated by stimulatory projections from the amygdala and inhibitory projections from the hippocampus. In addition, the PVN receives noradrenergic projections from the locus coeruleus (LC) and serotonergic projections from the raphe nuclei (RN). Stimulatory and inhibitory feedback loops are also represented, where cortisol is able to regulate its own synthesis and release by inhibiting ACTH and CRH synthesis in the pituitary and PVN respectively, and stimulating the hippocampus, which in turn may inhibit the PVN. Under repeated uncontrollable stress these feedback mechanisms result abolished, with the consequent hypercortisolism, alongside with increased reactivity of the amygdala and decreased activity of the hippocampus. Successful treatment is translated into recovery of these feedback mechanisms, with the consequent normalization of cortisol, decreased amygdala reactivity, increased hippocampal function, and normalization of noradrenergic and serotonergic systems.

Stress, and more specifically, early life stress, has been associated with the origin and development of depression and anxiety disorders (1–6). In this regard, it has been shown that chronic exposure to environmental stressors is followed by a set of adaptive responses, mediated by the activation of different neural structures involved in emotional and cognitive processing in the central nervous system (CNS), and the subsequent activation of the autonomic nervous system (ANS) and the hypothalamic-pituitary-adrenal axis (HPA) (7, 8). Environmental stressors are perceived and transmitted through sensory pathways to different structures in the CNS, including the thalamus and limbic areas, such as the amygdala and the hippocampus, and cortical areas, mostly located in the prefrontal cortex (PFC). Direct projections from the thalamus to the amygdala may provide primitive representations of stimuli, which in turn are potentiated by noradrenergic stimulation from the locus coeruleus (LC) to initiate a primary stress response. Indirect projections may also reach the amygdala from sensory and associative cortices, and transitional cortices, associated to the hippocampus (9). Hence, the hippocampus projects forward to the lateral nucleus of the amygdala, as well as the hypothalamic paraventricular nucleus (PVN), where it plays an inhibitory role (10, 11). The lateral nucleus of the amygdala projects to the basal, accessory basal, and central nuclei of the amygdala (CeA) (12). Therefore, the CeA projects to the lateral nucleus of the hypothalamus, which activates the sympathetic branch of the ANS (13), the dorsal motor nucleus of the vagus, which activates the para-sympathetic branch, and the PVN, therefore leading to the activation of the HPA axis (13, 14). Hence, the HPA axis may be activated through direct projections from the CeA, which project to the PVN (7), where the corticotropin releasing hormone (CRH, also termed corticotrophinreleasing factor, CRF) is synthesized and released into the hypophyseal portal blood to reach the anterior pituitary. CRH stimulates the transcription of the pro-opio-melanocortin (POMC) gene, a common precursor for adrenocorticotropic hormone (ACTH) and related peptides. ACTH is released into the bloodstream to reach the adrenal cortex, where it stimulates the biosynthesis and release of glucocorticoids, mainly cortisol (illustrated in **Figure 1**). These steroid hormones exert their effects through binding to mineralocorticoid receptors (MRs or type I) and glucocorticoid receptors (GRs or type II), constituting a hormone-receptor complex, which in turn may interact with specific DNA sequences located in the promoter region of target genes, termed glucocorticoid response element (GRE) (15), stimulating or inhibiting the expression of target genes. This has been described for the down-regulation of the POMC (16) and CRH genes (17), whereby cortisol is able to regulate its own synthesis and release through the negative feedback mechanisms that regulate HPA axis activity. In addition, cortisol may also down-regulate the HPA axis by binding to hippocampal GRs, which in turn inhibit the PVN, as well as exerting tonic inhibition through binding to hippocampal MRs (18, 19). During chronic stress these negative-feedback loops may be abolished, resulting in persistent activation of the HPA axis (15). Therefore, physiological rhythms characterized by wide diurnal variations, with morning zeniths and evening nadirs, are altered during chronic stress, which in turn may be translated into sustained increase in cortisol levels (1). In addition, chronic stress may also lead to decreased expression of brain derived neurotrophic factor (BDNF) in the hippocampus, which in turn may reduce its capability to inhibit the HPA axis (20, 21).These persistent alterations in the regulation of the HPA axis, such as the observed during chronic stress, has been associated with the origin and development of mood and anxiety disorders, where hyperactivity of the HPA axis, and the consequent hypercortisolism, represents one of the most consistent biological findings (6, 22, 23).

## The Role of the HPA Axis

Hyperactivity of the HPA axis is associated with increased synthesis and release of CRH from hypothalamic neurons in the PVN in response to stress. CRH-containing neurons have been also observed in other neural structures, such as the CeA (24, 25), which in turn activates the HPA axis through stimulatory projections to the PVN. In addition, reciprocal connections have been also observed between these CRH neurons and aminergic nuclei, including the LC and the raphe nuclei (RN) (4), therefore providing additional pathways for reciprocal interaction between the noradrenergic and the serotonergic systems, respectively, with the HPA axis during the stress response (4, 26) (illustrated in **Figure 1**). Thus, CRH neuronal circuits interact with the serotonergic and the noradrenergic systems, which are critically involved in mood and anxiety disorders (3). Moreover, CRH has been also associated with anxiety and encoding of emotional memories (3, 22) thus highlighting the critical role of the CRH system in the stress response and its role as an important factor in the long-lasting effects of stress, particularly regarding early life stressful experiences. In this regard, it has been shown that the impact of traumatic events during childhood represents a critical factor of vulnerability in the origin and development of mood and anxiety disorders later in life (4, 27, 28). The link between early adverse experiences, such as abuse, neglect or loss, and the development of mood and anxiety disorders has been shown to occur as a consequence of stressful conditions during different periods of life (29). Various studies focused on alterations in different limbic structures and the HPA axis. In this regard, it has been shown that exposure to early stressful events may lead to decreased availability and reduced efficacy of hippocampal GRs (27), which in turn may lead to glucocorticoid resistance and increased reactivity of the HPA axis in response to stressful situations later in life. Moreover, it has been shown that increased concentrations of cortisol along with decreased GRs induced by early stressful events were associated with decreased hippocampal function and volume in adulthood (30). Therefore, the impact of early adverse events may lead to long lasting changes, including hyper-reactivity of neural and neuroendocrine responses to stress, reflected in increased CRH, glucocorticoid resistance and reduced volume of the hippocampus (27, 31), all of which may contribute to shape potential responses to further stressful experiences later in life.

## The Role of the Serotonergic System

**Figure 2:**
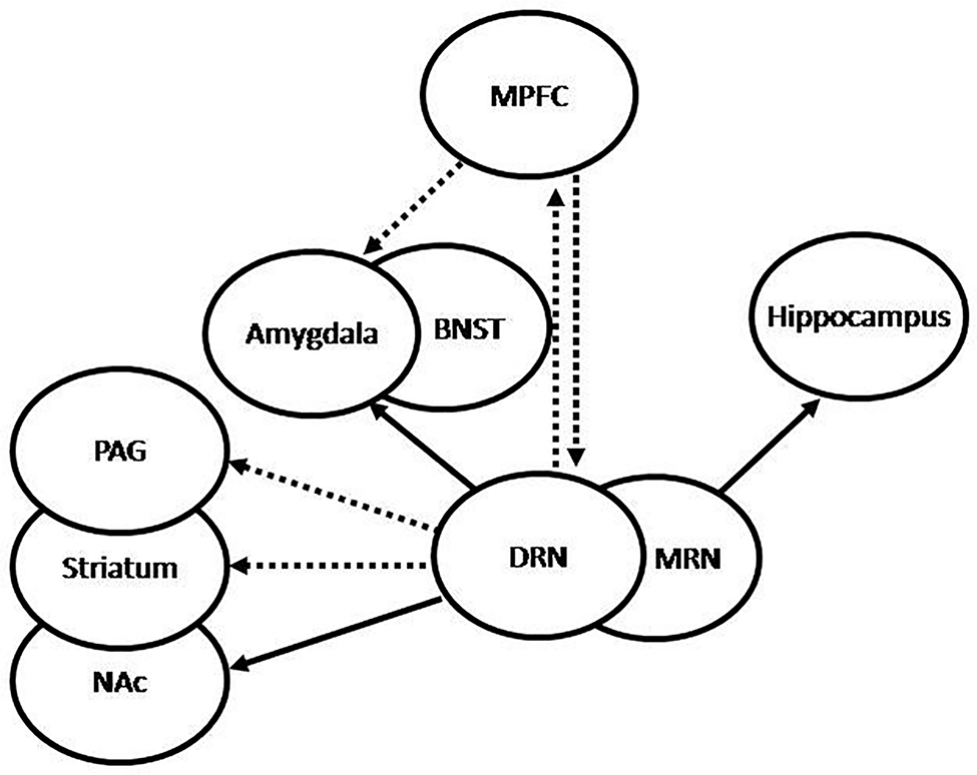
Graphic representation of 5HT projections from the raphe nuclei (RN). The dorsal RN (DRN) sends stimulatory projections to the central nucleus of the amygdala (CeA) and the bed nucleus of the stria terminalis (BNST), which participate in increased fear and anxiety, inhibitory projections to the periaqueductal grey (PAG) and the striatum, which participate in passive behavior, inhibitory projections to the medial prefrontal cortex (MPFC), and stimulatory projections to the nucleus accumbens (NAc), which participate in the regulation of complex behaviors and expression of emotions. Conversely, the MPFC sends inhibitory projections to the DRN and the amygdala, which may be translated into anti-anxiety effect. The medial RN (MRN) sends stimulatory projections to the hippocampus, which have been associated to increased tolerance to adverse stimuli and decreased anxiety. Under repeated uncontrollable stress the amygdala is increasingly stimulated by the DRN, which results in increased anxiety, the PAG and striatum are inhibited by the DRN, with the resulting passivity, and the serotonergic activation from the MRN to the hippocampus results impaired. Increased reactivity of the amygdala and decreased activation of the hippocampus may lead to increased activation of the hypothalamic-pituitary-adrenal (HPA) axis. Successful treatment is translated into recovery of these feedback mechanisms, with the consequent normalization of cortisol, decreased amygdala reactivity, increased hippocampal function, and normalization of the serotonergic systems.

It has been shown that deficient or altered serotonergic neurotransmission in the CNS plays a critical role in the origin and development of anxiety and depressive symptoms (26). The serotonergic system has its main sources in the RN, which project to diverse neural structures (illustrated in **Figure 2**). Serotonergic projections to the forebrain originate mainly in the dorsal (DRN) and medial RN (MRN) (32). The DRN-forebrain tract innervates various structures, many of them associated with anxiety-related and adaptive responses to stress (33–35), including the CeA (36), the bed nucleus of the stria terminalis (BNST) (37), the PVN, the nucleus accumbens (NAc), and certain areas of the PFC, particularly the medial PFC (MPFC) (38). In addition, the DRN also innervates structures related to regulation of fight-or-flight behavioral responses, such as the periaqueductal grey (PAG) (39, 40) and the striatum, which have been shown to be involved in passive coping behavior (41). Both neural structures, the PAG and the striatum, have also been associated with the state of anticipatory anxiety that plays a critical adaptive role in situations of danger, contributing to inform the amygdala about the current impact of negative experiences and the consequent emotional reactions (11). The MRN-forebrain tract projects to complementary neural structures, including the hippocampus and the hypothalamus (34, 42), and has been associated with tolerance to persistent aversive stimuli (43), such as those perceived during chronic stress, and adaptive control on negative emotional experiences (11). Thererfore, dysfunction of this system, particularly involving MRN-hippocampal projections, has been associated with decreased tolerance to aversive stimuli, learned helplessness, and subsequent depression (34). Serotonergic neurons in the RN have also been shown to interact with the noradrenergic and dopaminergic systems (44).

Serotonin (5-hydroxitryptamine, 5HT) released in the synaptic cleft binds to one or more of several 5HT receptors, classified as 5HT_1A−F_, 5HT_2A−C_; 5HT_3_, 5HT_4_, 5HT_5_, 5HT_6_, and 5HT_7_, most of them belonging to a family of G protein-coupled receptors (GPCRs), with the exception of the 5HT_3_ receptor, which is a ligand-gated ion channel (45). The 5HT_1A−F_ receptor family and the 5HT_5_ receptor couple with G_i_ protein, which inhibits adenylate cyclase (AC) activity, the 5HT_4_, 5HT_6_, and 5HT_7_ receptors couple with G_s_ protein, which stimulates AC activation, and the 5HT_2A−C_ receptor family couple with G_q_ protein, which stimulates the activity of phospholipase C (PLC) (46). In addition to the presence of different 5HT receptors, a critical role in the control of serotonergic neurotransmission is exerted by the serotonin transporter (5HTT), which acts to reuptake the remaining 5HT into the presynaptic terminal, therefore regulating the concentrations of the neurotransmitter in the synaptic cleft. The 5HTT represents the main target of various antidepressants, including the tricyclics (TCs) and the selective serotonin reuptake inhibitors (SSRIs). Blockade of the 5HTT by these drugs result in increased concentrations of 5HT in the synaptic cleft, leading in turn to increased activation of 5HT receptors (47). The efficacy of these antidepressants is associated with adaptive changes produced by its continuous administration, including desensitization or down regulation of somatodendritic 5HT_1A_ auto-receptors in the RN (48) and up-regulation of post-synaptic 5HT_1A_ (49), and desensitization of 5HT_2A_ receptors (50), mostly in the MRN-hippocampal tract. It has been shown that post-synaptic 5HT_1A_ receptors down-regulate or desensitize in different limbic structures by glucocorticoids or exposure to chronic stress (51–53). Cortisol may inhibit 5HT neurotransmission tonically through binding to MRs, while increased levels of cortisol, such as during chronically stressful conditions, bind predominantly to GRs, therefore interacting with GREs and inhibiting the expression of the 5HT1A gene (51). In addition, it has been shown that cortisol may exert a stimulatory effect on 5HT uptake *in vitro*, which has been attributed to an increased expression of the 5HTT gene by cortisol (54), further supporting the notion of reciprocal regulation between the HPA and 5HT systems, and their potential interactions in the interface between stress and depression.

## Effects of TCAs and SSRIs in the Regulation of the HPA Axis

A considerable number of patients suffering with chronic anxiety disorders exhibit hyperactivity of the HPA axis, with the consequent hypercortisolism. This has been described in patients with panic disorder (55) or generalized anxiety disorder (GAD) (56, 57), however, hypercortisolism in patients with GAD has not been observed in other studies (58, 59). Regarding posttraumatic stress disorder (PTSD), considerable evidence has also revealed alterations of the HPA axis (28). However, patients with PTSD exhibited decreased activity of the HPA axis, which has been attributed to exaggerated negative feedback or hypersecretion of CRH with consequent down-regulation of the anterior pituitary CRF receptors (60).

Successful pharmacological approaches often result in normalization of the HPA system. This led to further investigation of the role of the HPA axis in the pathophysiology of these disorders. Because different anti-anxiety agents, including tricyclic antidepressants (TCAs), selective serotonin reuptake inhibitors (SSRIs), and benzodiazepines (BZDs), have been demonstrated in some studies to normalize the HPA axis, various lines of research were developed focusing on the broader spectrum of mechanisms of action underlying the therapeutic effects of these agents.

It has been shown that TCAs and SSRIs, in addition to their well-known pharmacological effects, including blockade of neurotransmitter uptake and subsequent regulation of different pre- and post-synaptic receptors, may also induce significant changes in the HPA axis, associated with their therapeutic effects (61–69). Some of these, at least in part, have been attributed to the potential effect of anti-anxiety agents on transcriptional regulation of different molecules involved in the regulation of the HPA axis (70–74), including GRs, MRs, and CRH. In this regard, it has been proposed that altered GR gene regulation, which may be translated into diminished concentrations of GRs in different neural structures, more specifically hippocampal or hypothalamic GRs, may contribute to deficient feedback of the HPA system (63), which in turn may lead to the consequent alterations observed in patients suffering with depression or chronic anxiety disorders.

Increased GR mRNA expression has been initially observed *in vitro*, in cell cultures derived from hypothalamus or amygdala, upon incubation in the presence of desipramine or amytriptiline (70, 71). Similar results were also observed in studies in which chronic treatment with TCAs, but not short-term treatment, decreases CRH mRNA expression (61, 75, 76). Similar effects were also observed *in vivo* with long-term administration of imipramine. In this regard, it has been shown that long-term treatment with this TCA inhibited transcriptional regulation of the CRH gene, with the consequent decrease of CRH mRNA expression in the hypothalamus (76), which in turn resulted in a significant reduction in HPA axis activity (73, 76).

Regarding SSRIs, *in vitro* and *in vivo* studies demonstrated that long-term treatment with fluoxetine increased GR mRNA expression in hippocampal neurons (77, 78). More recently, *in vivo* studies demonstrated that long-term treatment with fluoxetine may also induce functional recovery of hippocampal GRs following chronic stress (79). Moreover, increased hippocampal GRs activation, including phosphorylation and subsequent nuclear translocation, was also observed after long-term treatment with fluoxetine, even in the absence of altered glucocorticoid secretion (79). Although these observations strongly suggest that this mechanism should be involved in the therapeutic effect of fluoxetine, more recent studies have also suggested that additional changes in GRs are not necessary for the behavioral efficacy of the SSRI (80).

It is noteworthy that GRs are expressed in the amygdala, particularly in the CeA (81), where glucocorticoids have been shown to stimulate the expression of CRH in this nucleus (82), in contrast to the inhibitory effect observed in the hypothalamic PVN (83). Cortisol up-regulation of CRH in the amygdala may be translated into activation of the whole system, because CRH projections from the CeA may exert stimulatory effect on the PVN, hence resulting in increased synthesis and release of CRH in the hypothalamus, with the consequent hyperactivity of the HPA axis. In this regard, overactivity of the amygdala represents another critical finding, frequently associated with depression and chronic anxiety disorders (84), which has been demonstrated in functional imaging studies (85–87). The amygdala has been shown to play a critical role in the physiopathology of anxiety and, as we mentioned previously, it is critically involved in the regulation of the HPA axis, more specifically, through CRH projections from the CeA, which stimulate the hypothalamic PVN (88). Because the amygdala represents one of the main sources of extra-hypothalamic CRH, hyper-activation of this limbic structure may be reflected in increased concentration of CRH in cerebrospinal fluid (CSF), as observed in many patients with depression (89–91), and elevated CRH transcript in animal models exposed to chronic stress conditions. Moreover, it was proposed that CRH overexpression in the CeA would be a main factor in the origin and development of depression (92). Therefore, a regulatory effect induced by SSRIs, translated into reduced GR and CRH gene expression in the CeA, may contribute, at least in part, to down regulation of the HPA axis, which is often observed with clinical improvement. In this regard, various studies were performed with escitalopram, which demonstrated that the SSRI was effective in the normalization of different physiopathological parameters related to HPA functioning. According to these studies, escitalopram was effective in reducing elevated concentrations of cortisol in patients with generalized anxiety disorder (GAD), which also was correlated with clinical improvement (93). In addition, escitalopram reversed the adverse effects of CRH overexpression in the CeA. *In vivo* studies also revealed that escitalopram was effective in reducing CRH expression in the hippocampus alongside increased GR expression in the hypothalamus and hippocampus, all associated with significant decreases in HPA axis reactivity (92). More recently, in a preclinical laboratory study, escitalopram inhibited expression of CRH and its receptors in the hypothalamus (94). The potential effects of TCAs and SSRIs in the regulation of the HPA axis may provide additional knowledge to better understand their therapeutic effects, although further research is necessary in this critical issue.

## Signaling Cascades Involved in the Regulation of Gene Expression

In order to understand the molecular mechanisms involved in the long lasting effects of TCAs and SSRIs, various studies were performed focusing on their regulatory effects on different components of the HPA axis. It has been suggested that long lasting effects of TCAs and SSRIs may involve up-regulation of the cAMP-mediated second messenger cascade, which in turn may lead to transcriptional regulation of different genes (95), including GR, CRH, and BDNF. Binding of different ligands to their specific GPCR are, of course, associated to second messenger cascades. In this regard, stimulation of Gs-coupled receptors may induce the activation of AC, with the consequent synthesis of cAMP, a second messenger responsible for the activation of cAMP-dependent protein kinase (PKA) (96). Therefore, stimulation of the AC-cAMP-PKA cascade may be translated into the activation of cAMP response element binding protein (CREB) (96, 97), which in turn operates as a transcription factor, mediating the effects of the cAMP cascade. In order to exert its regulatory effect, CREB should be activated, which is attained by phosphorylation at a single serine residue (Ser^133^) (96, 97). Once phosphorylated, CREB is able to regulate transcriptional events by binding to an enhancer element, located in the regulatory region of different genes, termed cAMP response element (CRE). It has been shown that phosphorylation of CREB may occur *via* activation of the AC-cAMP-PKA cascade and also *via* the calcium-dependent protein kinase (PKC) cascade, which allows CREB to act as a common downstream target of different stimuli, including those mediated by TCAs and SSRIs (67, 95, 98, 99). Molecular alteration of the AC-cAMP-PKA cascade has been described in patients suffering of depression (100), and various studies demonstrated that chronic treatment with different antidepressants contributed to repair this cascade at various molecular levels (101), therefore supporting the critical role played by CREB in the regulation of its target genes in the molecular mechanisms underlying the therapeutic effect of these molecules (102, 103). In this regard, it has been shown that the AC-cAMP-PKA cascade plays a critical role in the transcriptional regulation of CRH (104–107). Moreover, it has been shown that transcriptional activation of CRH depends on cAMP-PKA mediated phosphorylation of CREB, with the subsequent binding to CRE in the promoter region of the CRH gene (107). Similarly, there is considerable evidence for a cAMP-PKA mediated mechanism involved in GR regulation (108), therefore suggesting potential links between chronic treatment with TCAs and SSRIs, with the subsequent repairing effects on the AC-cAMP-PKA-pCREB cascade, and their regulatory effects on different components of the HPA axis at the transcriptional level.

Various *in vivo* studies demonstrated that chronic treatment with TCAs or SSRIs may up-regulate the expression of CREB in certain limbic regions (109). In this regard, *in vivo* studies revealed that chronic treatment with different antidepressants, including serotonin- and norepinephrine-selective reuptake inhibitors, led to increased expression of CREB mRNA in the hippocampus, particularly in CA1 and CA3 pyramidal cells and dentate gyrus granule cells (109, 110). In addition, BDNF mRNA expression was also increased in hippocampus after treatment with antidepressants (95), which suggests that chronic treatment with these molecules may lead to up-regulation of CREB, which in turn may increase the expression of different target genes, such as the BDNF gene (109), where a CRE has been described in its promoter region (111, 112). Therefore, up-regulation of CREB, with the subsequent increased expression of BDNF, may be critical to counteract the effects of stress on hippocampal neurons (30, 113). Moreover, it has been shown that BDNF participates in the regulation of the HPA axis (114, 115), which represents a potential link between the AC-cAMP-PKA molecular cascade, with the consequent activation of CREB and BDNF, with its regulatory effect on the HPA axis. Although, this molecular mechanism remains elusive and deserves further research to better understand the potential links between these molecular cascades. Because CREB has been associated with neuronal survival and plasticity in the hippocampus (116) and increased expression of CREB in hippocampus has been associated with therapeutic effects, it may represent a potential target for the design of novel therapeutic agents (110).

The therapeutic effect of TCAs and SSRIs has long been associated to up-regulation of 5HT_1A_ hetero-receptors (49, 117–119), which primarily work *via* G_i_, therefore inhibiting the AC-cAMP-PKA signaling cascade. Long-lasting effects of TCAs and SSRIs may involve activation of AC-cAMP-PKA, which in turn requires stimulation of GPCRs associated to G_s_, to activate this signaling cascade. In order to understand this apparent paradox, it is important to take into consideration that 5HT_1A_ hetero-receptors may activate indirect signaling mechanisms. Among these, it has been shown that activation of 5HT_1A_, associated to G_i_, may exert inhibitory effects on inhibitory pathways. In this regard, the effect of hippocampal 5HT_1A_ receptors, particularly in the dentate gyrus, has been associated to inhibition of GABAergic interneurons (120). Additional mechanisms have been also proposed, involving other GPCRs, which may also interact with 5HT_1A_–mediated signaling pathways. In this regard, 5HT_4_ receptors have been described in different neural structures, including the PFC, amygdala, and hippocampus (121). It has been shown that 5HT_4_ receptors are associated to G_s_ (122) and therefore are known to stimulate the AC-cAMP-PKA cascade, with the consequent phosphorylation of CREB. This, in turn, plays a critical role in the synthesis of BDNF, with the resulting facilitation of hippocampal neurogenesis (123, 124). In this regard, activation of 5-HT_4_ receptors have been also associated with the therapeutic effect of SSRIs (121, 125), therefore suggesting that potential interactions between 5-HT_1A_ and 5-HT_4_ receptors may be involved in the mechanism of action of antidepressants. Additional mechanisms have been also described involving potential interactions between 5-HT_1A_ and 5-HT_7_ receptors. In this regard, it has been demonstrated that different GPCRs may form homodimers and heterodimers, which may differ in various aspects with the non-associated GPCRs (126). It has been shown that 5HT_1A_ receptors may form heterodimers with others GPCRs, such as 5HT_7_, therefore resulting in different effects in comparison to the individual receptor by itself (127). In this regard, 5HT_7_ receptors have been widely described in different neural structures, including the hippocampus, hypothalamus, PFC, and amygdala (128, 129). The 5HT_7_ receptor is coupled to G_S_, therefore its activation results in stimulation of the AC-cAMP-PKA cascade (128). Heterodimerization of 5HT_1A_ and 5HT_7_ was observed *in vitro* (130), where it was shown that co-expression of both GPCRs decreased the activation of inhibitory G_i_, mediated by 5HT_1A_ receptors, without affecting the activation of stimulatory G_S_, mediated by 5HT_7_ receptors (130). Therefore, heterodimerization of both GPCRs may lead to important functional changes in their downstream signaling, with the consequent regulatory effects (131).

## Effects of BZDs in the Regulation of the HPA Axis

The HPA axis is also regulated by other neurotransmitters, including γ-aminobutyric acid (GABA), the major inhibitory neurotransmitter in the CNS (132). It has been shown to be closely involved in the regulation of hypothalamic function (133–135). Inhibitory GABAergic input has been shown to innervate hypophysiotropic CRH neurons in the medial parvocellular hypothalamic PVN (136, 137) through direct input from peri-PVN sources or indirectly from diverse limbic structures. Direct GABAergic projections may reach the PVN from adjacent hypothalamic nuclei and the BNST (138) or indirectly from various cortical and limbic structures, including the hippocampus, through the ventral subiculum, the amygdala, and the PFC, particularly the ACC, prelimbic, and infralimbic areas (137). Local GABAergic projections to the PVN may in turn be activated or inhibited by glutamatergic or GABAergic projections from cortical and limbic areas, which are closely involved in adaptive responses to stress and, therefore in the regulation of the HPA axis (139). The inhibitory role of the hippocampus in the regulation of the HPA axis is therefore, in part, mediated by GABAergic projections from the ventral subiculum to the PVN, which may allow hippocampal processing, including information related to previous experiences and to the current context, exert adaptive influence on the stress response (137).

At the molecular level, GABAergic effects in the CNS are mediated by two types of postsynaptic receptors, GABA-A and GABA-B (140). The GABA-A receptor is a complex, constituted by diverse sub-units, with specific binding sites for its natural ligand, GABA, benzodiazepines (BZDs), and barbiturates. Upon binding of GABA, activation of postsynaptic GABAA receptors allows the opening of specific chloride (Cl^-^) ion channels, with the resulting influx of Cl^-^ and the consequent hyperpolarization of postsynaptic neurons and inhibition of cell firing. Binding of BZDs to their specific site on the GABA-A receptors enhances the binding of GABA to its specific binding-site in the same receptor, which leads to increased frequency of Cl^-^ channels with the resulting hyperpolarization and the consequent inhibition of target neurons (140, 141). Therefore, BZDs represent a family of anti-anxiety agents, whose mechanism of action has been associated to their enhancement of GABAergic function in different areas of the CNS, including their potential role in the modulation of the HPA axis, particularly in those patients suffering with depression and anxiety disorders. In this regard, the effect of alprazolam, a potent BZD agonist, has been studied *in vivo*, where it was observed that both GABA and the BZD exerted inhibitory effect on the HPA axis (141) and, according to previous studies, this effect was attributed to the effect of BZDs on GABAergic receptors inhibiting the CRH system (142). The presence of GABA-A receptors in the hypothalamus further supports this central mechanism of action. Interestingly, diazepam, another well known BZD, have been shown to decrease corticosterone levels *in vivo* (143) and it has been shown that BZDs decrease cortisol levels in healthy volunteers and depressed patients in a dose-dependent manner (141, 142).

This regulatory effect was further studied *in vivo* with alprazolam, where it was observed that the BZD was capable of inhibiting the HPA axis, and this was attributed to the effect of the BZD on CRH neurons, which may contribute to its therapeutic efficacy (144). The potential role for CRH in the pathophysiology of anxiety disorders was extensively studied (145), therefore proposing further research on the effects of anti-anxiety agents on CRH neurotransmission. In this regard, it has been shown that acute treatment with alprazolam decreased CRH concentrations in the LC (146). Moreover, the effect of alprazolam was further studied *in vivo*, where it was shown to decrease CRH concentrations in the LC after acute or chronic administration (147). The LC receives a rich CRH innervation (148) contains CRH receptors (149) and is critically involved in the pathophysiology of stress and anxiety disorders (150). Therefore, the effects of alprazolam on hypothalamic CRH neurons, are likely both direct and indirect through the LC (151). More recently, *in vivo* studies with lorazepam and clonazepam demonstrated that both BZDs were effective to reversing anxiety-like behavior, including social-avoidance, and these effects were correlated with their inhibitory effect on the HPA axis, mediated by suppression of CRH activity (152). Moreover, it has been shown that both BZDs were effective to reducing stress-induced CRH mRNA expression in the hypothalamus (152).

According to the molecular mechanisms previously described in the aforementioned section, it has been demonstrated that anti-anxiety agents, including BZDs, as well as TCAs and SSRIs, may exert certain effects on the HPA axis. However, although this may provide further information to better understand the molecular mechanisms involved in their therapeutic effects, it has been shown that these effects only account for a small part of their therapeutic and pharmacological effects, which may be reflected in the partial improvement in the hypercortisolism observed in certain patients suffering depression or anxiety disorders.

## Conclusion

The role of the HPA axis in the pathophysiology of depression and chronic anxiety disorders has been extensively studied, including the particular role played by their different components, including CRH neurotransmission, cortisol, and their specific receptors, and the genes coding for each of these molecules. It remains unclear, however, how important the effects of anti-anxiety agents on the HPA axis activity are in mediating their therapeutics benefits and moreover whether further modulation of CRH and related systems might augment our currently available agents.

## Author Contributions

Both authors contributed equally.

## Conflict of Interest

CN's disclosures: Research/Grants: National Institutes of Health (NIH); Consulting (last three years): Xhale, Takeda, Taisho Pharmaceutical Inc.,Signant Health, Sunovion Pharmaceuticals Inc., Janssen Research & Development LLC, Magstim, Inc., Navitor Pharmaceuticals, Inc., TC MSO, Inc., Intra-Cellular Therapies, Inc., EMA Wellness, Gerson Lehrman Group (GLG), Acadia Pharmaceuticals; Stockholder: Xhale, Celgene, Seattle Genetics, Abbvie, OPKO Health, Inc., Antares, BI Gen Holdings, Inc., Corcept Therapeutics Pharmaceuticals Company, TC MSO, Inc., Trends in Pharma Development, LLC, EMA Wellness; Scientific Advisory Boards: American Foundation for Suicide Prevention (AFSP), Brain and Behavior Research Foundation (BBRF), Xhale, Anxiety Disorders Association of America (ADAA), Skyland Trail, Signant Health, Laureate Institute for Brain Research (LIBR), Inc.; Board of Directors: Gratitude America, ADAA, Xhale Smart, Inc.; Income sources or equity of $10,000 or more: American Psychiatric Publishing, Xhale, Signant Health, CME Outfitters, Intra-Cellular Therapies, Inc., Magstim, EMA Wellness; Patents: Method and devices for transdermal delivery of lithium (US 6,375,990B1) Method of assessing antidepressant drug therapy *via* transport inhibition of monoamine neurotransmitters by ex vivo assay (US 7,148,027B2) Compounds, Compositions, Methods of Synthesis, and Methods of Treatment (CRF Receptor Binding Ligand) (US 8,551, 996 B2).

The remaining author declares that the research was conducted in the absence of any commercial or financial relationships that could be construed as a potential conflict of interest.
